# RNA-sequencing analysis of lung primary fibroblast response to eosinophil-degranulation products predicts downstream effects on inflammation, tissue remodeling and lipid metabolism

**DOI:** 10.1186/s12931-017-0669-8

**Published:** 2017-11-10

**Authors:** Stephane Esnault, Ksenija Bernau, Elizabeth E. Torr, Yury A. Bochkov, Nizar N. Jarjour, Nathan Sandbo

**Affiliations:** 1 Department of Medicine, Division of Allergy, Pulmonary and Critical Care Medicine, The University of Wisconsin-Madison School of Medicine and Public Health, K4/928 Clinical Science Center MC 9988, 600 Highland Avenue, Madison, WI 53792 USA; 2 Department of Pediatrics, University of Wisconsin School of Medicine and Public Health, Madison, WI 53792 USA

**Keywords:** Fibroblast, Eosinophil, Il-3, IgG, Degranulation, Lung, RNA-sequencing, Inflammation, Tissue remodeling, Lipid metabolism

## Abstract

**Background:**

The association of eosinophils with inflammation and tissue remodeling is at least partially due to their release of toxic granule proteins and other mediators, including cytokines. Tissue remodeling and consequent functional defects are affected by activity of connective tissue fibroblasts. Exaggerated fibroblast activation, accumulation and change of phenotype may lead to fibrosis and loss of tissue function. So far, little information has been reported on how eosinophils affect inflammation and tissue remodeling via the activation of fibroblasts. We have recently shown that eosinophil activation with IL-3 led to a robust eosinophil degranulation on immunoglobin-G (IgG) coated plates. Thus, in the present study, we analyze the effects of IL-3-activated eosinophil degranulation products on primary human lung fibroblasts (HLF) using whole transcriptome sequencing.

**Methods:**

Conditioned media was obtained from eosinophils that were pre-activated with IL-3 or IL-5 and subsequently cultured for 6 h on IgG to induce degranulation. This conditioned media was added on human lung fibroblasts (HLF) for 24 h and the cell lysates were then subjected to whole transcriptome sequencing to identify global changes in gene expression. Differentially expressed genes were analyzed using the Ingenuity Pathway Analysis (IPA), and validated by qPCR.

**Results:**

In HLF, the expression level of 300 genes was changed by conditioned media from IL-3-activated eosinophils compared to control fibroblast cultures. Among these 300 genes, the expression level of 35 genes coding for known proteins was upregulated by IL-3- versus IL-5-pre-activated eosinophils. Of the 35 upregulated genes, IPA identified *C3*, *CH25H*, *CXCL1*, *CXCL8*, *CYP1A1*, *ICAM1*, *IL6* and *UCN2* as having downstream functions on inflammation, tissue remodeling and lipid synthesis. This analysis combined with previous RNA sequencing analyses of eosinophils suggest IL-1ß, OSM and TNFSF12 as potential upstream regulators of fibroblasts.

**Conclusions:**

This study has identified several novel pro-inflammatory and pro–remodeling mediators produced by fibroblasts in response to activated eosinophils. These findings may have significant implications on the role of eosinophil/fibroblast interactions in eosinophilic disorders.

**Electronic supplementary material:**

The online version of this article (10.1186/s12931-017-0669-8) contains supplementary material, which is available to authorized users.

## Background

Allergic inflammation is characterized by a type-2 immune response. Mast cells and eosinophils are critical for the development of early- and late-phase type-2 immune response, respectively. Eosinophilia has been associated with chronic asthma symptoms and severity [1–4]. Evidence suggests that eosinophils contribute to asthma pathogenesis via the release of cytotoxic granule proteins and other mediators, including cytokines and lipids [5, 6]. The release of these mediators from eosinophils leads to tissue damage and recruitment/activation of other cell types, including fibroblasts.

Asthma is marked by airway inflammation and remodeling. The structural changes of airway remodeling result in airflow limitation and are associated with increased asthma severity [7]. Airway remodeling includes epithelial changes, angiogenesis, and increased smooth muscle mass, and activation of fibroblasts to differentiate into pro-inflammatory-producing fibroblasts and extracellular matrix (ECM)-producing myofibroblasts [8].

Eosinophils are an important source of growth factors and cytokines that can influence fibroblast function and airway remodeling. Transforming growth factor-β (TGF-ß) and interleukin-1 (IL-1) are two cytokines that have been implicated in airway remodeling, and they are known to be derived from eosinophils [9–15]. TGF-β can activate fibroblasts to differentiate into myofibroblasts and to produce pro-fibrotic proteins [10, 16, 17]. IL-1 also can induce airway remodeling [18], but interestingly IL-1 and TGF-ß may have opposing effects on the differentiation of fibroblasts, as TGF-ß-induced fibroblast differentiation can be attenuated by IL-1, particularly in lung fibroblasts [19–21]. Moreover, TGF-ß and IL-1, associated with the eosinophil granule major basic protein (MBP), can induce expression of the pro-fibrotic and inflammatory IL-6 family genes (IL-6, IL-11, and leukemia inhibitory factor) [22]. Human blood eosinophils co-cultured with skin fibroblasts leads to secretion of IL-6, CXCL1, and CCL2 from fibroblasts independently of cell contact, indicating that soluble mediators are responsible for activation of these genes in fibroblasts [23, 24]. Therefore, while the role of eosinophils on the fibroblast biology has been previously studied and reported, the expression level of only few genes has been examined.

Eosinophil-driven pathology involves the release of toxic proteins and mediators involved in inflammation and remodeling, via degranulation or piecemeal release. Because high amounts of immunoglobulin G (IgG) are present in airways [25, 26], eosinophil degranulation on IgG has been used as a physiologically relevant model to analyze the mechanisms of eosinophil degranulation [27, 28]. We have recently reported that IL-3-pre-activated eosinophil strongly degranulated on heat aggregated human IgG (HA-IgG), releasing ~25% of their total cellular eosinophil-derived neurotoxin (EDN) in 6 h [29]. In the present study, we have used this eosinophil degranulation model [29] to examine the function of the endogenic eosinophil compounds on primary human lung fibroblasts (HLF). To understand the global effects of eosinophil mediators on fibroblasts, we performed whole-transcriptome RNA sequencing (RNA-seq) analysis of gene expression in cultured HLF. We hypothesized that fibroblast response to eosinophil-degranulation products would lead to potential downstream effects on tissue inflammation and remodeling.

## Methods

### Subjects, cell preparations and cultures

Eosinophil isolation was performed under a study protocol approved by the University of Wisconsin-Madison Health Sciences Institutional Review Board. Informed written consent was obtained from subjects prior to participation. Peripheral blood eosinophils were obtained from allergic subjects with (*n* = 2) and without mild asthma (*n* = 1). Subjects with prescriptions for low doses of inhaled corticosteroids did not use their corticosteroids the day of the blood draw.

Eosinophils were purified by negative selection as previously described [29] from three female donors with 0.12, 0.2 and 0.47 × 10^6^ eosinophils per ml of whole blood. Briefly, 200 ml of heparinized blood was diluted 1:1 in HBSS and was overlaid above Percoll (1.090 g/ml). After centrifugation at 700 x*g* for 20 min, at room temperature, the mononuclear cells were removed from the plasma/Percoll interface and erythrocytes were eliminated from the cell pellet by hypotonic lysis. The remaining pellet was resuspended in 2% new calf serum in HBSS. Cells were then incubated with anti-CD16, anti-CD3, anti-CD14 and anti-Glycophorin-A beads from Miltenyi (San Diego, CA), and run through an AutoMACS (Miltenyi). Eosinophil preparations with purity > 99% and viability ~98% were used the same day, ~5 h after the blood draw.

Human lung fibroblasts (HLF) were isolated as described previously [30, 31] using de-identified tissue samples from thoracic surgical resection specimens. These samples were collected and characterized in collaboration with the biobanking services run by the Carbone Cancer Center Translational Science BioCore at the University of Wisconsin-Madison, under Institutional Review Board approval. To obtain non-fibrotic fibroblasts, we utilized adjacent (uninvolved) lung from lobectomy or biopsy specimens from patients undergoing lung resection for pulmonary nodules and who did not have any identifiable lung disease by history or histologic assessment. All specimens used for fibroblast isolation were examined by a pathologist to ensure that no underlying lung disease (emphysema, idiopathic lung diseases, etc.) was present on histology. Briefly, to isolate fibroblasts, tissue specimens were placed in DMEM with 100 units/ml streptomycin, 250 ng/ml amphotericin B, 100 units/ml penicillin, and 10 μg/ml ciprofloxacin. Alveolated lung tissue was minced and plated onto 10-cm plates in growth medium containing DMEM supplemented with 10% FBS, 2 mM L-glutamine, and antibiotics, as above. Expanded populations of fibroblasts were subsequently subcultured after 4–5 days, resulting in the development of a homogenous fibroblast population. All primary cultures were used from passages 5–10 and maintained on tissue culture plastic until the time of experiments. Two different HLF lines from different donors were used in this study.

### Preparation of eosinophil-conditioned media

Peripheral blood eosinophils were cultured at 1 × 10^6^/ml in medium (RPMI 1640 plus 10% fetal bovine serum (FBS)) with IL-3 (2 ng/ml) or IL-5 (4 ng/ml) for 20 h. After 20 h stimulation with cytokines, eosinophils were washed and suspended at 1 × 10^6^/ml in fresh medium (no cytokine), and 1 ml was added to a 24-well plate, which had been coated overnight with HA-IgG (10 μg/ml; 350 μl/well) and saturated with 0.1% gelatin for 30 min at 37 °C. Heat aggregation of human IgG (HA-IgG) was performed in PBS for 30 min at 63 °C, as previously described [29]. IL-3-preactivated eosinophils were also seeded on uncoated (no HA-IgG) wells, as negative control for eosinophil degranulation. After a 6 h incubation, the three types of supernatant fluids (conditioned media: IL-3, IL-3 on HA-IgG (IL3IgG) and IL-5 on HA-IgG (IL5IgG)) were harvested and stored at −80 °C, before analysis by ELISA for EDN release (degranulation), and before addition on cultured HLF. Human EDN ELISA (MBL, Woburn, MA) has a minimum detection limit of 0.62 ng/ml.

### Fibroblasts cultured with eosinophil-conditioned media

HLF were treated in 1:1 dilution of maintenance media (DMEM/10% FBS, 2 mM L-glutamine, and antibiotics as above) and eosinophil-conditioned media. The three types of eosinophil-conditioned media described above (IL-3, IL-3 on HA-IgG (IL3IgG) and IL-5 on HA-IgG (IL5IgG)) were obtained from three different blood donors. Each conditioned medium was tested individually on the HLF from both lung biopsy donors. Controls included HLF cultured in medium only (RPMI with 5% FBS) and HLF cultured with 1 ng/ml plus 0.5 μg/ml of HA-IgG (rhIL3IgG). This latter control was used to account for 1) potential release of intracellular IL-3 during IL-3-activated eosinophil degranulation, and 2) any release of coated HA-IgG during the 6 h degranulation period. After 24 h, HLF were harvest with RLT extraction buffer from Qiagen for total RNA extraction.

### RNA preparation and RNA-seq analysis

Total RNA was extracted from HLF using the RNeasy Mini Kit (Qiagen, Valencia, CA) and treated with DNase (RNase-free DNase kit, Qiagen). Fifteen μl of total RNA were recovered with concentrations ranging from 70 to 180 ng/μl and with ratios 260/280 nm ranging from 1.98 to 2.04. RNA samples were submitted to the University of Wisconsin-Madison Biotechnology Center (Madison, WI) for RNA quality and integrity evaluation via Agilent 2100 Bioanalyzer platform (Agilent Technologies) and whole-transcriptome sequencing. The sequencing library from mRNA was prepared using TruSeq Stranded mRNA Library Preparation Kit (Illumina; San Diego, CA, USA) and RNA-seq (1 × 100 bases) was carried out using Illumina HiSeq 2500 platform. SeqMan Ngen (v.13) and ArrayStar with Qseq module (v.13) software packages (DNAStar, Madison, WI) were used to map sequence reads to human reference genome (GRCh38), apply statistical analyses and identify global gene expression changes for both fibroblast donors. Of note, the two fibroblasts (L20 and L21) were analyzed separately, and only genes up- or down-regulated by more than 1.5 fold for both L20 and L21 were considered in our analyses. Raw data and normalized RPKM (Reads Per Kilobase per Million mapped reads) values have been deposited to the NCBI Gene Expression Omnibus (GEO) database and assigned accession number (GSE102860). Differentially expressed genes were analyzed using DAVID Bioinformatics Resources 6.8(Beta) (National Institute of Allergy and Infectious Diseases (NIAID), NIH) [32] and the Ingenuity Pathway Analysis (IPA) web-based software (Qiagen) to identify the relevant biological mechanisms and pathways.

### Real-time quantitative (q) PCR

The reverse transcription reaction was performed using the Superscript III system (Invitrogen/Life Technologies, Grand Island, NY, USA). Gene expression levels were determined by qPCR using SYBR Green Master Mix (SABiosciences, Frederick, MD, USA) or Taqman. For PCR using SYBR Green, forward and reverse specific primers (see Additional file 1: Table E1 for primer sequences) were designed using Primer Express 3.0 (Applied Biosystems, Carlsbad, CA, USA) and blasted against the human genome to determine specificity using http://www.ncbi.nlm.nih.gov/tools/primer-blast. The reference gene primers, ß-glucuronidase ((GUSB), forward: caggacctgcgcacaagag, reverse: agcgtgtcgacccattc), was used to normalize the samples. Standard curves were performed and efficiencies were determined for each set of primers. Efficiencies ranged between 91 and 96%. Data are expressed as fold change using the comparative cycle threshold (∆∆CT) method as described previously [29].

### Statistical analyses

Statistical analyses of the pathways, potential upstream regulators and downstream functions of the genes changed by the IL3IgG conditioned media in HLF were generated by the Ingenuity Pathway Analysis (IPA) package from Qiagen. Statistical analyses of the qPCR were performed using the SigmaPlot 11.0 software package (Systat Software, Inc., San Jose, CA, USA). One Way ANOVA followed by the Holm-Sidak analysis were used to compare the three groups (IL3, IL3IgG and IL5IgG), and *p* < 0.05 was considered statistically significant.

## Results

### Generation of conditioned media from degranulated eosinophils

**Fig. 1:**
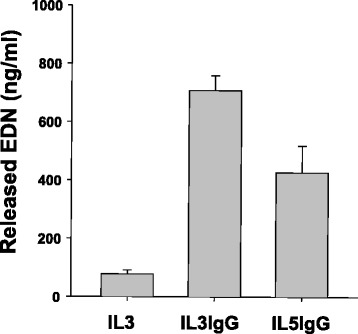
Evaluation of eosinophil degranulation by the measurement of EDN in eosinophil conditioned media. To prepare the eosinophil conditioned media that will be tested on human lung fibroblasts, human blood eosinophils were activated with IL-3 or IL-5 for 20 h and were then added on heat-aggregated (HA) human serum IgG for 6 h. Three conditioned media were prepared, including two conditioned media from IL-3-activated eosinophils, cultured for 6 h on coated HA-IgG (IL3IgG) or uncoated wells (IL3). The third conditioned medium was from IL-5-activated eosinophils seeded for 6 h on HA-IgG (IL5IgG). EDN released from eosinophils in the three conditioned media was measured by ELISA. The graph is an average ± SEM of three different cultures from three different eosinophil donors. The three groups were statistically different from each other as determine using the One-Way Analysis of Variance followed by the Holm-Sidak method (*p* < 0.001, *n* = 3)

Eosinophils activated for 20 h with IL-3 or IL-5 were added on coated heat-aggregated (HA)-human serum IgG for 6 h (IL3IgG and IL5IgG). The morphology of the eosinophils pre-activated with IL-3 or IL-5 and seeded on coated HA-IgG is shown on Additional file 2: Figure E1, where pictures of cultures were taken at 2 h, 4 h and 6 h. Additional file 2: Figure E1 suggests that in the earlier time-points, eosinophils adhered to HA-IgG. However, the adhesion was reversible for IL-5-activated eosinophils while IL-3 activation led to cell breaks and release of free granules (Additional file 1: Figure E1). As a negative control for eosinophil degranulation, IL-3-activated eosinophils were seeded in uncoated wells (IL3). In this condition, (no IgG), eosinophils do not adhere to the bottom of the wells (not shown). Conditioned media were harvested 6 h after eosinophils from three donors were seeded on IgG. As shown on Fig. 1, IL3IgG eosinophils released ~15-fold more EDN than the control IL3 (seeded on uncoated wells), and ~2-fold more than IL5IgG. These conditioned media samples were diluted (2-fold) in serum free medium, and added to human lung primary fibroblasts (HLF) for 24 h before RNA expression analyses by next-generation RNA sequencing (RNA-Seq) and RT-qPCR.

### RNA-seq analyses of HLF cultured with eosinophil conditioned media

For RNA-seq analyses, total RNA was extracted from 2 HLF cell lines (L20 and L21) cultured for 24 h with conditioned media from eosinophils (from *n* = 2 different donors) degranulated on HA-IgG after a 20 h priming with either IL-3 (IL3IgG) or IL-5 (IL5IgG). Also, HLF culture controls included HLF cultured with medium only or with 1 ng/ml of IL-3 plus 0.5 μg/ml of HA-IgG (rhIL3IgG) to account for the possible release of rhIL-3 from eosinophils degranulating on HA-IgG, and for the possible detachment of coated IgG during degranulation.

In a first analysis, we wished to examine gene expression levels changed in HLF by the degranulation products from IL-3-activated eosinophils. For this purpose, gene expression by fibroblasts incubated with IL3IgG conditioned media (mean values of two donors) was compared to both control fibroblast cultures (medium and rhIL3IgG). Genes whose expression level is changed in the same direction (up or down) by more than 1.5 fold in both HLF lines (L20 and L21) were included in this first dataset. Among these genes, 169 genes were upregulated (Additional file 3: Table E2). Analysis using DAVID Bioinformatics Resources identified 24 genes coding for secreted proteins (including 10 cytokines) and 47 genes coding for membrane proteins (Table 1). Additionally, 131 genes were downregulated (Additional file 4: Table E3), among them, 44 were genes coding for membrane proteins, 23 coded for nuclear proteins, while only 10 coded for secreted proteins.

**Table 1:** Genes upregulated by >1.5-fold in both HLF lines (L20 and L21) at 24 h, by degranulated products from eosinophils pre-activated with IL-3 for 20 h and seeded on coated HA-IgG (IL3IgG) for 6 h, from two different eosinophil donors, compared to HLF controls cultured with medium only, and with rhIL-3 (1 ng/ml) plus soluble HA-IgG (1 μg/ml)

Secreted Proteins	Apolipoprotein A-I (**APOA1**); bone morphogenetic protein 8b (**BMP8B**); C-C motif chemokine ligand 2 (**CCL2**); chemokine (C-C motif) ligand 4-like 2 (**CCL4L2**); chondroadherin like (**CHADL**); collagen type IV alpha 3 (**COL4A3**); complement component 1, q subcomponent-like 1 (**C1QL1**); complement component 3 (**C3**); complement component 8 alpha subunit (**C8A**); C-X-C motif chemokine ligand 1 (**CXCL1**); C-X-C motif chemokine ligand 8 (**CXCL8**); fibroblast growth factor 10 (**FGF10**); fibronectin leucine rich transmembrane protein 1 (**FLRT1**); gremlin 2, DAN family BMP antagonist (**GREM2**); interleukin 32 (**IL32**); interleukin 6 (**IL6**); Klotho (**KL**); mannosyl (alpha-1,3-)-glycoprotein beta-1,4-Nacetylglucosaminyltransferase, isozyme A (**MGAT4A**); nodal growth differentiation factor (**NODAL**); platelet factor 4 variant 1 (**PF4V1**); plexin domain containing 1 (**PLXDC1**); secreted and transmembrane 1 (**SECTM1**); SPARC like 1 (**SPARCL1**); urocortin 2 (**UCN2**)
Membrane proteins	ADAM metallopeptidase domain 20 (**ADAM20**); ATPase phospholipid transporting 8B4 (**ATP8B4**); bone marrow stromal cell antigen 2 (**BST2**); carnitine palmitoyltransferase 1B (**CPT1B**); CD177 molecule (**CD177**); cholesterol 25-hydroxylase (**CH25H**); complement component 8 alpha subunit (**C8A**); chromosome 1 open reading frame 204 (**C1orf204**); chromosome 2 open reading frame 82 (**C2orf82**); cytochrome P450 family 1 subfamily A member 1 (**CYP1A1**); cytochrome P450 family 1 subfamily B member 1 (**CYP1B1**); dachsous cadherin-related 2 (**DCHS2**); fibronectin leucine rich transmembrane protein 1 (**FLRT1**); G protein-coupled receptor 17 (**GPR17**); galactose-3-O-sulfotransferase 2 (**GAL3ST2**); glutamyl aminopeptidase (**ENPEP**); guanine nucleotide binding protein (G protein), gamma 3 (**GNG3**); immunoglobulin superfamily member 6 (**IGSF6**); intercellular adhesion molecule 1 (**ICAM1**); interleukin 12 receptor subunit beta 1 (**IL12RB1**); Janus kinase 3 (**JAK3**); Klotho (**KL**); mannosyl (alpha-1,3-)-glycoprotein beta-1,4-N-acetylglucosaminyltransferase, isozyme A (**MGAT4A**); NPC1-like 1 (**NPC1L1**); NTPase, KAP family P-loop domain containing 1 (**NKPD1**); nucleoporin 210 kDa (**NUP210**); PAPPA antisense RNA 1 (**PAPPA-AS1**); par-6 family cell polarity regulator beta (**PARD6B**); plexin domain containing 1 (**PLXDC1**); potassium voltage-gated channel modifier subfamily S member 2 (**KCNS2**); protein tyrosine phosphatase, receptor type N2 (**PTPRN2**); RAB9B, member RAS oncogene family (**RAB9B**); receptor (chemosensory) transporter protein 4 (**RTP4**); reticulon 1 (**RTN1**); secreted and transmembrane 1 (**SECTM1**); shroom family member 2 (**SHROOM2**); solute carrier family 1 member 3 (**SLC1A3**); solute carrier family 16 member 6 (**SLC16A6**); solute carrier family 18 member A2 (**SLC18A2**); solute carrier family 22 member 14 (**SLC22A14**); solute carrier family 51 alpha subunit (**SLC51A**); sortilin related VPS10 domain containing receptor 2 (**SORCS2**); synapse differentiation inducing 1 (**SYNDIG1**); TBC1 domain family member 3H (**TBC1D3H**); TMEM110-MUSTN1 readthrough (**TMEM110-MUSTN1**); V-set and immunoglobulin domain containing 10 like (**VSIG10L**); zinc finger CCCH-type containing 12A (**ZC3H12A**)
Nuclear proteins	Apolipoprotein A-I (**APOA1**); brain expressed X-linked 2 (**BEX2**); calmodulin like 6 (**CALML6**); cell death inducing DFFA like effector c (**CIDEC**); cilia and flagella associated protein 46 (**CFAP46**); distal-less homeobox 1 (**DLX1**); ETS homologous factor (**EHF**); family with sequence similarity 71 member E1 (**FAM71E1**); fibroblast growth factor 10 (**FGF10**); forkhead box D2 (**FOXD2**); hes family bHLH transcription factor 1 (**HES1**); histone cluster 1, H2bi (**HIST1H2BI**); histone cluster 2, H2be (**HIST2H2BE**); histone cluster 2, H3d (**HIST2H3D**); inhibitor of CDK, cyclin A1 interacting protein 1 (**INCA1**); LIM homeobox 4 (**LHX4**); NFKB inhibitor alpha (**NFKBIA**); NFKB inhibitor zeta (**NFKBIZ**); par-6 family cell polarity regulator beta (**PARD6B**); phosphatase domain containing, paladin 1 (**PALD1**); SRY-box 7 (**SOX7**); ubiquitin specific peptidase 17-like family member 1 (**USP17L1**); vitamin D (1,25-dihydroxyvitamin D3) receptor (**VDR**); v-rel avian reticuloendotheliosis viral oncogene homolog B (**RELB**); zinc finger CCCH-type containing 12A (**ZC3H12A**); zinc finger MYND-type containing 15 (**ZMYND15**); ZNF559-ZNF177 readthrough (**ZNF559-ZNF177**)

The differentially expressed genes (up- (Additional file 3: Table E2) and down-regulated (Additional file 4: Table E3)) were analyzed using the Ingenuity Pathway Analysis (IPA) web-based software. The Predictive Causal Analytic tool identified three major and highly overlapping Regulator Effect Networks. These three networks contained part of the 300 genes present in our dataset (target molecules), potential upstream regulators of these genes, and the association between these genes with potential downstream biological functions (Table 2). More than 15% (27) of the 169 genes upregulated in our dataset were part of the target genes grouped into the three networks. There was 85% similarity among the target genes between the three networks. Around 80% of the potential identified upstream regulators (Table 2) were common among the three networks, and more than 65% of the projected functional downstream effects of these genes were similar between the three networks as well. Therefore, the aggregate of all three networks is shown in Table 2. The main predicted downstream functions of the fibroblast genes were related to the immune response, tissue remodeling and lipid metabolism (Table 2).

**Table 2:**
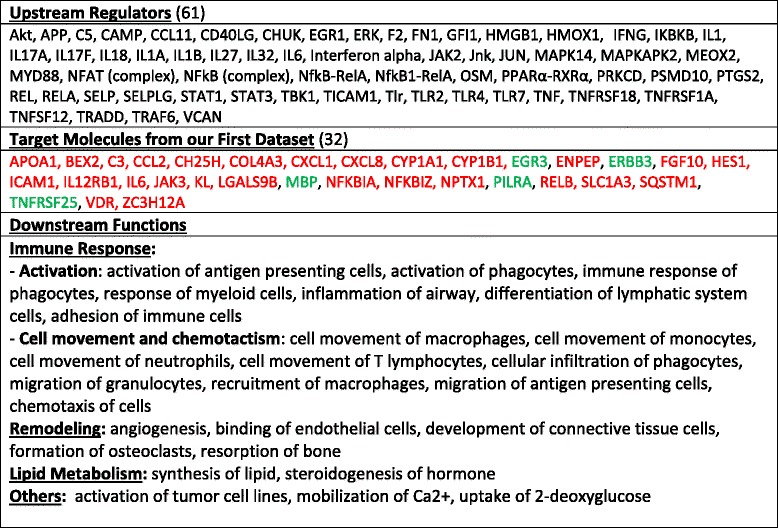
Regulator Effect Networks identified by Ingenuity Pathway Analysis (IPA) using the 300 genes of our first dataset (Additional file 3: Table E2 and Additional file 4: Table E3). Summation of all three identified networks

Genes  in our dataset (Additional file 1: Table E1 and Additional file 3: Table E2)

The upstream regulators are molecules that are predicted to activate or inhibit the expression of the genes in our dataset, according to the literature compiled in the Ingenuity® database. IPA determined the predicted overlap between an upstream regulator and the genes listed in our dataset. Additionally, IPA uses a z-score algorithm to make statistical prediction for activation or inhibition of a specific upstream regulator to genes present in our dataset. As shown in Table 3, the main significant groups of upstream regulators are composed of cytokines, kinases, intracellular signaling molecules and transcription regulators. The upstream regulators with the four highest z-scores for each group is shown in Table 3. Furthermore, other potential significant upstream regulators are listed on Additional file 5: Table E4. Of note and as expected, kinase inhibitors for MAPK, NF-kB, PI3K display negative z-scores predicting inhibitory effects on the target genes (Additional file 4: Table E3). In addition to these potential upstream regulators shown in Table 3, Additional file 4: Table E3 and Additional file 5: Table E4, miR-223 (z-score = −2.0; *p* = 8.51 × 10^−3^) was also found to be a potential upstream down-regulator of *ABCA13*, *AK8* and *EGR3* (Additional file 6: Table E6). This is particularly interesting in the light of a recent report by Zangari et al. where it is shown that neutrophils release miR-223-3p via extracellular vesicles [33]. Notably also, miR-223 expression was increased in bronchoalveolar lavage cells enriched in airway eosinophils 48 h after a segmental allergen challenge [34]. Subsequently, we tested the validity of these upstream regulators by analyzing the expression of the upstream regulator receptors on HLF. For this purpose, we extracted and listed the expression level of receptors for the upstream regulators predicted by IPA from our RNA-seq database. This is shown in Table 2, Table 3 and Additional file 4: Table E3. As shown in Additional file 7: Table E5, HLF expressed relatively high levels of the receptors for TNF-α, IL-1, OSM, IL-6, IFN-γ, IL-17A, IL-17F, IL-27, IFN-α, TNFSF12, C5 and F2. Of note, while *IL2RG* (common γ-chain receptor) is not expressed, IL-4 can signal via its type II receptor, whose subunits *IL4R* and *IL13RA1* are both expressed by HLF (Additional file 7: Table E5). In addition to *IL2RG*, the receptors for GM-CSF and IL-18 were not expressed in HLF, in any of our conditions (Additional file 7: Table E5). Notably also, while IL-3-activated eosinophils may be the source of these upstream regulators, HLF might also release some of these regulators during the 24 h culture with eosinophil conditioned media. Therefore, the potential upstream regulators that are extracellularly localized and expressed by HLF were listed in Additional file 7: Table E5. HLF express *IL6*, *TNFSF12*, *C5*, *IL32*, *FN1*, *APP*, *HMGB1* and *TLR4* (Additional file 7: Table E5), which could all participate in HLF activation in an autocrine way. Eosinophils can produce and/or store a variety of cytokines and other ligands [35]. While we have not yet performed transcriptome and proteome of IL-3-activated eosinophils, we have recently reported RNA-seq data using resting human blood eosinophils [36]. In that study, among the potential upstream regulators identified by IPA, transcripts coding for CAMP (LL37), CHUK, HMGB1, HMOX1, IL-1, IL-18, IL-32, OSM, SELPG and TNFSF12, were detected in eosinophils. Therefore, altogether, our data indicate that IL-1ß, OSM (oncostatin-M), IL-32 and TNFSF12 (Tweak) may be candidates to explain the direct effect of conditioned medium from degranulated eosinophils on HLF. Interestingly, *IL32* was upregulated in fibroblasts by IL3IgG conditioned media (Additional file 7: Table E5), suggesting that IL-32 could function endogenously on fibroblasts.

**Table 3:** Upstream Regulators of the genes listed in our dataset (target genes, Additional file 3: Table E2 and Additional file 4: Table E3) as predicted by IPA analysis

Upstream regulator	*P* value of overlap	Activation z-score	Target genes listed in our dataset	
			Genes upregulated	Genes downregulated
Cytokine				
TNF	*2.86 10* ^*−4*^	*3.788*	APOA1, BST2, C3, CCL2, CH25H, CIDEC, COL4A3, CPT1B, CXCL1, CXCL8, CYP1A1, CYP1B1, DLX1, EHF, FGF10, HES1, ICAM1, IL32, IL6, KL, KYNU, LGALS9B, NFKBIA, NFKBIZ, RELB, SLC1A3, SQSTM1, VDR, ZC3H12A (29)	
IL1B	*2.61 10* ^*−3*^	*3.059*	C1R, C3, CCL2, CXCL1, CXCL8, CYP1A1, EHF, HES1, ICAM1, IL32, IL6, LGALS9B, NFKBIA, NFKBIZ, RELB, SLC1A3, VDR, ZC3H12A (18)	
OSM	*7.71 10* ^*−3*^	*2.853*	C1R, CCL2, CH25H, CXCL1, CXCL8, CYP1B1, ICAM1, IL32, IL6, SLC16A6, VDR (11)	ERBB3
IL6	*1.15 10* ^*−2*^	*2.824*	APOA1, BST2, C3, CCL2, CXCL1, CXCL8, CYP1A1, CYP1B1, ICAM1, IL6, NFKBIA, USP17L1 (12)	TNFRSF25, TLR1
Signaling				
NFkB (complex)	*1.20 10* ^*−3*^	*3.746*	BEX2, C1R, C3, CCL2, CXCL1, CXCL8, EHF, ICAM1, IL32, IL6, KYNU, NFKBIA, NFKBIZ, RELB, ZC3H12A (15)	
MYD88	*3.31 10* ^*−7*^	*3.081*	C3, CCL2, CH25H, CXCL1, CXCL8, FGF10, ICAM1, IL32, IL6, NFKBIA, NFKBIZ, RELB, ZC3H12A (13)	PILRA
Akt (group)	*1.00 10* ^*−2*^	*2.574*	CCL2, CXCL8, HES, IL32, IL6, KL	ERBB3
ERK (group)	*9.60 10* ^*−3*^	*2.559*	CCL2, CXCL8, CYP1A1, ICAM1, IL6, KYNU, NFKBIZ	
Transcription Factor				
JUN	*1.08 10* ^*−4*^	*3.097*	CCL2, CXCL1, CXCL8, CYP1B1, HES1, ICAM1, IL6, NFKBIA, NFKBIZ, PARD6B, RELB, VDR (12)	MBP, PAK3,
RELA	*3.55 10* ^*−5*^	*2.863*	BEX2, C3, CCL2, CXCL1, CXCL8, CYP1A1, EHF, FGF10, HES1, ICAM1, IL32, IL6, NFKBIA, RELB (14)	
STAT1	3.05 10^−2^	2.574	C3, CCL2, CH25H, CXCL8, HES, ICAM1, IL6	
STAT3	1.67 10^−2^	2.439	BEX2, BST2, CCL2, CXCL8, ICAM1, IL12RB1, IL6, NFKBIZ, SLC1A3, VDR	EGR3

**Fig. 2:**
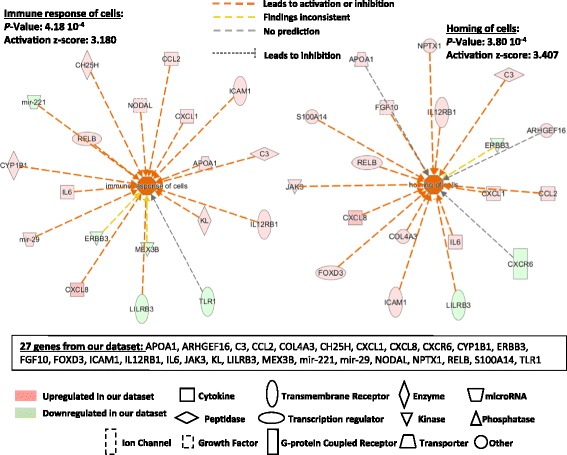
IPA Downstream Analysis as Networks identified functions on the immune response. The association between genes of our dataset #1 (300 genes) and specific downstream functions received an overlap *p* value and a *z*-score for prediction of activation or inhibition on the functions: “immune response of cells” and “homing of cells”. Twenty-seven genes from our dataset either upregulated or downregulated in HLF by IL3IgG eosinophil conditioned media, are involved in the regulation of “immune response of cells” and “homing of cells” with a significant z-score toward increased “activation”

**Fig. 3:**
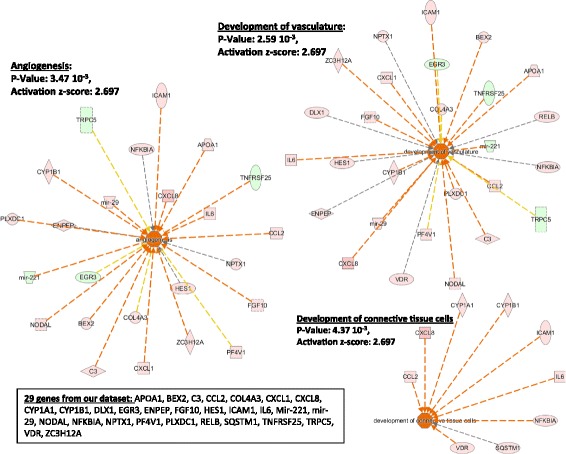
IPA Downstream Analysis as Networks identified functions on tissue remodeling. The association between genes of our dataset #1 (300 genes) and specific functions received an overlap *p* value and a *z*-score for prediction of activation or inhibition on the functions: “angiogenesis” and “development of vasculature” and “development of connective tissue cells”. Twenty-nine genes from our first dataset either upregulated or downregulated in HLF by the IL3IgG eosinophil conditioned media, are involved in the regulation of these three functions with a significant *z*-score toward increased “activation”

**Fig. 4:**
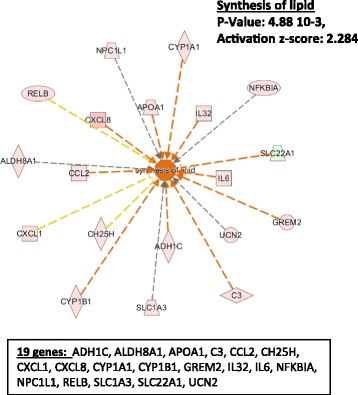
IPA Downstream Analysis as Networks identified functions on lipid metabolism. The association between genes of our dataset #1 (300 genes) and specific functions received an overlap *p* value and a *z*-score for prediction of activation or inhibition on the function: “synthesis of lipid”. Nineteen genes from our first dataset either upregulated or downregulated in HLF by the IL3IgG eosinophil conditioned media, are involved in the regulation of this function with a significant *z*-score toward increased “activation”

The downstream analysis performed by IPA shows a variety of downstream functions affected by the proteins coded by the genes composing our dataset. As shown in Table 2, downstream functions of 32 genes from our dataset as defined by the Regulator Effect Networks have been regrouped in three major categories: immune response, tissue remodeling and lipid metabolism. Identical to the Regulator Effect Networks (Table 2), another IPA tool called, Downstream Analysis as Networks, identified two major downstream networks, which are linked to the immune response (Fig. 2). These two networks (immune response of cells and homing of cells) are predicted to be regulated by 27 of the 300 genes present in our dataset (Additional file 1: Table E1 and Additional file 3: Table E2), with relatively high positive z-scores (3.180 and 3.407; Fig. 2). In addition to these two networks, angiogenesis, development of vasculature and development of connective tissue cells, are three major mechanistic networks characterizing tissue remodeling (Fig. 3). These three mechanistic networks include as many as 29 genes from our dataset (~10%). Finally, a third mechanistic network, named synthesis of lipid, is affected by 19 genes of our dataset and is illustrated on Fig. 4.

IPA network algorithm also generates networks showing interactions between the genes of our dataset in relation with other neighboring genes present in a Global Molecular Network. Thirty-five total genes, or group of genes, are present in each network. Fifteen networks were formed using > 10 genes from our dataset. The networks with the two highest scored (*p*-value < 10^−23^) and including > 15 genes/groups from our dataset are shown on Additional file 8: Figure E2 and Additional file 9: Figure E3. These 15 genes or gene products are directly or indirectly associated with the protein kinase B (PKB or Akt) (Additional file 8: Figure E2) or the c-jun N-terminal protein kinase (JNK) and TGF-ß (Additional file 9: Figure E3), and mostly characterized by the inflammatory response, cell movement and cell/tissue development. Of note, the functional networks created by IPA analysis (Additional file 8: Figs. E2 and Additional file 9: Figure E3) suggest that there is no known tight functional connection either between the 27 genes characterizing “inflammation” (Fig. 2), or between the 29 genes characterizing “tissue remodeling” (Fig. 3) or between the 19 genes characterizing “lipid metabolism” (Fig. 4). Although it is worth noticing that the network 1 (Additional file 8: Figure E2) includes *IL12RB1, mir-221, mir-29* and *NODAL* that are all part of the 27 genes related to inflammation. In addition, the network 2 (Additional file 9: Figure E3) includes *APOA1, C3, COL4A3, CYP1B1* and *FGF10* that are part of tissue remodeling, and *ADH1C, APOA1, C3* and *CY1B1* that are all part of the genes characterizing lipid metabolism. This indicates that there are some known functional relationships between genes characterizing “Inflammation”, “tissue remodeling” or “lipid metabolism” (Figs. 2, 3 and 4).

In a second analysis of our RNA-seq database, we restricted the list of genes obtained in the first dataset by eliminating the genes which expression levels were not changed in HLF cultured with IL-3- compared to IL-5-activated eosinophils (IL3IgG versus IL5IgG). For this purpose, gene expression by HLF incubated with IL3IgG conditioned media (average of two donors) for 24 h was compared to fibroblast incubated with IL5IgG conditioned media (average of two donors), and to both controls HLF (medium and rhIL3IgG). Genes whose expression level changed by more than 1.5-fold in the same direction (up or down) in both HLF lines are included in this second restricted dataset. In these comparisons, 35 genes coding for known proteins were upregulated (Table 4), and analysis using DAVID Bioinformatics Resources 6.8 identified eight genes coding for secreted proteins (also shown in Table 1). In addition, in this dataset #2, 37 genes were downregulated (Additional file 6: Table E6), among them, three were genes coding for secreted proteins. Table 4 and Additional file 6: Table E6 give a brief description of the 72 (35 up +37 down) genes and their relationship with the networks created by IPA using the 300 genes in our dataset #1 (Tables 2 and 3; Figs. 2, 3 and 4, Additional file 2: Figure E1 and Additional file 8: Figure E2). Furthermore, Additional file 10: Table E7 shows the genes of our dataset #2 associated with the six most significant global molecular networks formed by IPA (Additional file 8: Figure E2, Additional file 9: Figure E3 and not shown). The genes of our dataset #2, which are part of the three downstream functional categories (immunity, remodeling and lipid metabolism, Figs. 2, 3 and 4), are listed in Table 5. The eight genes shown in Table 5 are involved in at least one of these three categories, and four genes (*C3, CXCL1, CXCL8, IL6*) are part of all three categories. From Table 5, *CXCL1, CXCL8, ICAM1* and *IL6* were arbitrarily selected to validate our RNA-seq analyses by RT-qPCR.

**Table 4:** Genes upregulated by IL3IgG- versus IL5IgG-activated eosinophil conditioned medium and versus control media (medium only and rhIL3IgG) in fibroblasts

Gene	Brief description	Regulators-networks (IPA analyses)
BMP8B (secreted)	Bind TGF-ß receptors– Activates SMAD	Network 1: inflammatory response (Additional file 2: Figure E1)
C3 (secreted)	Modulates inflammation – Antimicrobial activity	Target molecule (Table 2); Regulated by TNF, IL1B, IL6, NFkB, MYD88, RELA, STAT1 (Table 3); Networks: immune response, remodeling and lipid metabolism (Figs. 2, 3, 4); Network 2: cell trafficking (Additional file 8: Figure E2)
C8A (secreted)	Antimicrobial activity	
CH25H	Cholesterol-Lipid metabolism	Target molecule (Table 2); Regulated by TNF, OSM, STAT1 (Table 3); Networks: immune response, lipid metabolism (Figs. 2 and 4)
CHADL (secreted)	Inhibits collagen fibrillogenesis	
CIDEC	Insulin sensitivity	Regulated by TNF (Table 3); Network 1: inflammatory response (Additional file 2: Figure E1)
CPT1B	Lipid transport	Regulated by TNF (Table 3)
CXCL1 (secreted)	Chemoattractant for neutrophils – Tumor growth	Target molecule (Table 2); Regulated by TNF, IL1B, OSM, IL6, NFkB, MYD88, JUN, RELA (Table 3); Networks: immune response, remodeling and lipid metabolism (Figs. 2, 3, 4)
CXCL8 (secreted)	Neutrophil attraction and activation	Target molecule (Table 2); Regulated by TNF, IL1B, OSM, IL6, NFkB, MYD88, Akt, ERK, JUN, RELA, STA1, STA3 (Table 3); Networks: immune response, remodeling and lipid metabolism (Figs. 2, 3, 4)
CYP1A1	Cholesterol and lipid synthesis	Target molecule (Table 2); Regulated by TNF, IL1B, ERK, RELA (Table 3); Networks: remodeling and lipid metabolism (Figs. 3, 4)
EGLN2 (egl-9 family hypoxia-inducible factor 2)	Hypoxia tolerance-Tumor proliferation	
FLRT1	Fibroblast growth factor	Regulated by tretinoin (Additional file 4: Table E3)
FOXD2	Somatogenesis	
FOXD3	Promotes development of neural cells	Regulated by tretinoin (Additional file 4: Table E3); Networks: immune response (Fig. 2)
FRMPD1 (FERM and PDZ domain containing 1)	Stabilizes membrane-bound GPSM1	
GNG3	Gamma subunit of guanine nucleotide binding proteins	
ICAM1	Leukocyte migration-Rhinovirus receptor	Target molecule (Table 2); Regulated by TNF, IL1B, OSM, IL6, NFkB, MYD88, ERK, JUN, RELA, STAT1, STAT3 (Table 3) Networks: immune response, remodeling (Figs. 2,3)
IGSF6		
IL6 (secreted)	Induces inflammatory response-Insulin resistance-Nerve cells differentiation	Target molecule (Table 2); Regulated by TNF, IL1B, IL6, NFkB, MYD88, Akt, ERK, JUN, RELA, STAT1, STAT3 (Table 3) Networks: immune response, remodeling, lipid metabolism (Figs. 2, 3, 4)
KLKB1 (kallikrein B, plasma (Fletcher factor) 1	Blood coagulation-Inflammation	
KRT36 (keratin 36, type I)	Type I hair keratin	
MYO1H (myosin IH)	Actin based intracellular movement	
NATD1 (N-acetyltransferase domain containing 1)		
NFKBIZ	LPS induced-Increases IL-6 production	Target molecule (Table 2); Regulated by TNF, IL1B, MYD88, ERK, JUN, STAT3 (Table 3)
NUP210	Part of nuclear pore complex	
OR51B5		
PALD1		
PCBP3 (poly(rC) binding protein 3)	RNA-binding proteins	
PRODH (proline dehydrogenase (oxidase) 1)	Mitochondrial proline degradation	Network 1: inflammation, neurological disease (Additional file 2: Figure E1)
PYHIN1 (pyrin and HIN domain family, member 1)	Promote ubiquitination- IFN-induced	Network 2: cell trafficking (Additional file 8: Figure E2)
SOX7	Represses Wnt/beta-catenin pathway	
SYNDIG1	IFN-induced- Synaptogenesis	
TTC6 (Tetratricopeptide repeat protein 6)		
UCN2 (secreted)	Homeostasis after stress	Networks: lipid metabolism
ZC3H12A	CCL2-induced- Cell death- mRNA decay (IL-6 and IL12B)- Inhibits miRNAPromote IL-4-induced M2- Increase glial differentiation	Target molecule (Table 2); Regulated by TNF, IL1B, NFkB (Table 3); Networks: remodeling (Fig. 3)
ZMYND15	Spermatogenesis- transcriptional repressor via histone deacetylase

**Table 5:** Genes in dataset #2 part of the three downstream function categories defined by IPA using dataset #1 (Table 2, Figs. 2, 3 and 4)

Downstream function categories (Figs. 2, 3 and 4)	Genes	
	Upregulated	Downregulated
Immune response	C3, CH25H, CXCL1, CXCL8, ICAM1, IL6	ERBB3, TLR1
Remodeling	C3, CXCL1, CXCL8, CYP1A1, ICAM1, IL6	
Lipid synthesis	C3, CH25H, CXCL1, CXCL8, CYP1A1, IL6, UCN2	

**Fig. 5:**
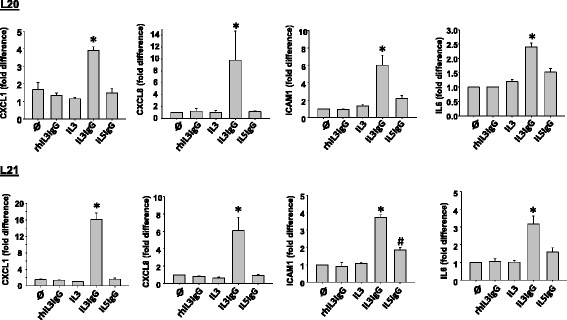
PCR analysis demonstrates upregulation of* CXCL1*, *CXCL8*, *IL6* and *ICAM1* expression levels in HLF activated with conditioned media from eosinophils pre-activated with IL3 and degranulating on aggregated IgG. Conditioned media were prepared from eosinophils pre-activated for 20 h with IL-3 or IL-5, and seeded on heat-aggregated human IgG for 6 h (IL3IgG or IL5IgG). Conditioned media from eosinophils pre-activated with IL-3 and seeded in uncoated wells (IL3) were also prepared. HLF were cultured for 24 h with the three types of conditioned media (IL3IgG, IL5IgG and IL3), a control medium (Ø) and medium including 1 ng/ml of recombinant human IL-3 plus 0.5 μg/ml of HA-IgG (rhIL3IgG). RT-qPCR were performed from total mRNA extracted from two HLF lines (L20 and L21) cultured in the five different conditions. For each HLF lines, controls (Ø) and rhIL3IgG are *n* = 2. IL3, IL3IgG and IL5IgG conditioned media were prepared from three different eosinophil donors, including two donors previously used for the RNAseq analyses. IL3, IL3IgG and IL5 were compared using One Way Anova (*n* = 3), and * indicates that IL3IgG is upregulated compared to IL3 and IL5IgG; and # indicates that IL5IgG is upregulated compared to IL3

For RT-qPCR analysis, conditioned media (IL3IgG, IL5IgG and IL3 (no IgG)) from all three eosinophil donors were used on the two HFL lines. Controls included fibroblasts cultured in medium only and fibroblasts cultured with 1 ng/ml of IL-3 plus 0.5 μg/ml of HA-IgG (rhIL3IgG). In agreement with the RNA-seq data, qPCR data (Fig. 5) confirms that *CXCL1*, *CXCL8, ICAM1* and *IL6* are all significantly upregulated by IL3IgG conditioned media compared to IL5IgG and controls.

## Discussion

Our study shows that expression level of ~300 genes is changed in cultured human lung primary fibroblasts (HLF) by conditioned media obtained from degranulated eosinophils. Among these genes, the Ingenuity Pathway Analysis (IPA) identified at least 66 genes known to be associated with either inflammation (immune response, homing of cells), tissue remodeling (angiogenesis, vasculature, connective tissue development), or lipid synthesis. Furthermore, among this first dataset of 300 genes, we have found a unique gene set that characterizes the activation of fibroblasts by conditioned media from IgG-driven eosinophil degranulation after pre-activation with IL-3 compared to a pre-activation with IL-5. In this gene set, we found differential expression of 72 genes (dataset #2) coding for known proteins. According to IPA, among these 72 genes, eight of the 35 up-regulated genes, *C3*, *CH25H*, *CXCL1, CXCL8, CYP1A1, ICAM1, IL6* and *UCN2* are connected to inflammation, tissue remodeling or lipid synthesis, with *C3, CXCL1, CXCL8* and *IL6* being part of all three categories.

Increased expression levels of *IL6*, *CXCL1* and *CXCL8* have previously been observed in co-culture experiments of human blood eosinophils and fibroblasts [23, 24]. In those studies, IL-6 secretion by fibroblasts was dependent on p38, ERK and NF-kB; and CXCL1 and CXCL8 inhibitions were reached by blockade of p38 and ERK [24]. In agreement with this previous publication, as identified by IPA, all these intracellular mediators (kinases and NF-kB) were significant potential upstream activators of the genes upregulated in our datasets.

C3, CXCL1, CXCL8, ICAM1 and IL-6 are all enhancers of neutrophil activation and trafficking [37–41], suggesting that one effect of IL-3-dependent activation of eosinophils is enrichment in soluble mediators that can influence fibroblasts to participate in the recruitment of a neutrophilic immune response. Although the role of the protein product of C3 (C3a) on neutrophils remains controversial [42], it has been previously reported that mice lacking C3 exhibit less neutrophil infiltration in brain injury and infection models [43]. Notably, the reduction of inflammatory cell, including neutrophil, recruitment in these C3 knock out mice, was probably at least partially due to the ability of C3a to activate endothelial cells to produce adhesion membrane receptors and to exhibit stress fiber formation [44, 45]. In addition to its role in neutrophilia, C3a can induce smooth muscle cell contraction [46, 47], participates in mast cell degranulation [48] and increases production of pro-inflammatory cytokines by monocytes [49]. Therefore, in addition to IL-6, CXCL1 and CXCL8 (IL-8), C3 is another factor produced by HLF in the presence of eosinophils that can mediate inflammation and remodeling.

Beyond enhancement of the neutrophilic recruitment, we also found increased production of mediators that can participate in promoting inflammation or activate fibroblasts in a paracrine/autocrine fashion. One such gene is *CH25H*. *CH25H* codes for an enzyme, cholesterol 25-hydroxylase, a multi-transmembrane endoplasmic reticulum protein, which produces the oxysterol, 25-hydroxycholesterol (25-HC) from cholesterol. The addition of the second hydroxyl group at position 25 makes 25-HC much more hydrophilic than cholesterol. Furthermore, CYP7B1 can convert 25-HC into 7α, 25-HC, which as an EBI2 (GPR183) ligand triggers intracellular signaling, and increases cell migration, particularly B cells and eosinophil [50, 51]. Interestingly, 25-HC is increased in the airway of COPD patients, and correlates positively with neutrophilic inflammation and negatively with pulmonary function [52]. Finally, 25-HC increases the production and release of TGF-ß by fibroblasts and subsequently the production of markers of myofibroblasts, including α-smooth muscle actin, collagen 1 and metalloproteinases [53], a finding that we have confirmed in our lab (data not shown). Therefore, *CH25H* and its product, 25-HC have a role in tissue remodeling. In our model, we speculate that fibroblast-derived *CH25H* could potentiate myofibroblast differentiation via the production of 25-HC.

One interesting signature found by IPA analyses suggest that the genes upregulated in HLF by EOS-conditioned medium enhanced angiogenesis. Inflammation and angiogenesis are thought to be closely interdependent processes (reviewed in [54]). One of the feature of asthma pathophysiology is the development of microvascular network in lungs, with severe asthma displaying higher vascularization compared to moderate and mild asthma [55]. In asthma, increased vasculature reduces airway compliance and increases airway narrowing [56]. Therefore, EOS-activated HLF may lead to increased airway remodeling via vasculature development.

Besides inflammation and remodeling, the downstream potential function associated with lipid synthesis is also intriguing. UCN2 (urocortin-2) is one molecule linked to synthesis of lipids by IPA analysis. The receptor for UCN2, CRF2 may play an important role in mechanisms that control food intake and energy homeostasis [57–59]. Xiong Y et al. have recently reported that UCN2/UCN3 via CRF2 are responsible for hypoxia-induced lipolysis and loss of weight via a cAMP/PKA pathway in white adipose tissue [60].

The differential gene expression signature that we found in HLF cultured with conditioned media from IL-3- compare to IL-5-pre-activated eosinophils may be explained by higher eosinophil degranulation level ([29]; Fig. 1). In addition to increased degranulation, there may be differential protein production in IL-3- versus IL-5-activated eosinophils. We have recently reported that compared to GM-CSF and IL-5, IL-3 activation led to higher protein translation and production of semaphorin-7A and the low-affinity receptor for IgG (Fc-gamma-RII, CD32) [29, 61, 62]. Therefore, we speculate that compared to IL-5, eosinophils pre-activated with IL-3 further produce a subset of proteins that are potential upstream regulators of fibroblasts. IPA analysis identified a list of ~60 upstream potential regulators. We cross-referenced this list with gene expression data from our current RNA-seq and found that HLF primary lines express relatively high levels of receptors for 12 of these potential upstream regulators, including TNF-α, IL-1, OSM (oncostatin-M), IL-6, IFN-γ, IFN-α, TNFSF12 (tweak), C5 and F2 (thrombin). Release of soluble cytokines such as IL-1ß could account for many of the gene expression changes that we observed, as several studies have shown that eosinophils are a source of IL-1ß [11–15]. Furthermore, previous co-culture experiments using neutralizing antibodies have demonstrated that anti-IL-1ß inhibited ~70% of eosinophil-induced IL-6 release by fibroblasts while anti-TGF-ß, −IL-1α and -TNF-α had lesser inhibitory effects (~20%) on IL-6 release [23]. While eosinophils are a source of TGF-ß [9, 10], surprisingly TGF-ß was absent from the potential upstream regulators in our analyses. In accordance with the lack of TGF-ß in the upstream regulators, no major genes coding for markers of myofibroblast and ECM proteins were identified as induced as part of our RNA-seq datasets. Thus, it is possible that this model of degranulation on IgG does not allow piecemeal degranulation of TGF-ß. However, it is worth noting that TGF-ß was part of a downstream Global Molecular Network (Additional file 9: Figure E3) along with collagen proteins, suggesting that fibroblast-derived autocrine or paracrine effects may ultimately lead to production of ECM components. Future studies in our lab will be needed to assess the components of eosinophil-derived media that mediate our observed effects.

One limitation of analyzing global gene expression changes using RNA-seq is the potential lack of correlation between transcript and protein levels due to the effects of regulation of translation, as we have previously shown for several eosinophil genes [29, 62]. Future studies that use techniques such as high-resolution mass spectroscopy proteomic technologies will help to resolve these limitations.

## Conclusions

Our study has identified the gene signature in human primary lung fibroblasts that results from soluble factors released by IL-3-activated eosinophils. Global analyses indicate that a major downstream effect of this exposure is an enhancement of the expression of several genes that could promote granulocytic inflammation (*C3, CXCL1, CXCL8, ICAM1* and *IL6*). In addition, components of this novel gene signature predict regulation of tissue remodeling, angiogenesis, and lipid metabolism. This work suggests that exposure to eosinophil-derived mediators may promote fibroblasts participation in neutrophil recruitment and airway remodeling.

## Additional files

Additional file 1: Table E1. Primer sequences used for real-time PCR. (DOCX 15 kb)

Additional file 2: Figure E1. IL-3-pre-activated eosinophils strongly adhere to coated HA-IgG and ultimately release free granule proteins. Human blood eosinophils were activated with IL-3 or IL-5 for 20 h and were then added on heat-aggregated (HA) human serum IgG. Photomicroscopy of IL-3 and IL-5-pre-activated eosinophils on HA-IgG was performed at 2 h, 4 h and at 6 h, using a digital camera from Olympus. (PDF 223 kb)

Additional file 3: Table E2. Genes (169) up regulated by >1.5 fold in both fibroblasts lines (L20 and L21) cultured with IL3IgG eosinophil conditioned media (average of 2 eosinophil donors), compared to HLF cultured in medium only, and cultured with rhIL-3 plus HA-IgG. (PDF 88 kb)

Additional file 4: Table E3. Genes (131) down regulated by >1.5 fold in both fibroblasts lines (L20 and L21) cultured with IL3IgG eosinophil conditioned media (average of 2 eosinophil donors), compared to HLF cultured in medium only, and cultured with rhIL-3 plus HA-IgG. (PDF 88 kb)

Additional file 5: Table E4. Upstream Regulators of the genes listed in our dataset (target genes, Additional file 3: Table E2 and Additional file 4: Table E3) as predicted by IPA analysis. (PDF 141 kb)

Additional file 6: Table E6. Genes downregulated by IL3IgG- versus IL5IgG-activated eosinophil conditioned medium and control media (medium only and rhIL3IgG) in fibroblasts. (PDF 94 kb)

Additional file 7: Table E5. HLF (L20 and L21) expression levels for the receptors of the upstream regulators identified by IPA (Tables 2 and 3, and Additional file 4: Table E3), as determined by our RNA-seq analysis. (PDF 92 kb)

Additional file 8: Figure E2. Global Molecular Network #1 generated by IPA downstream analysis. Interactions between the genes of our dataset #1 (300 genes) in relation with other neighboring genes present in a Global Molecular Network. Thirty-five total genes or group of genes are present in each network. This network characterized genes related to the inflammatory response and development disorder. It includes 15 genes and 1 group from our dataset. (PDF 321 kb)

Additional file 9: Figure E3. Global Molecular Network #2 generated by IPA downstream analysis. Interactions between the genes of our dataset #1 (300 genes) in relation with other neighboring genes present in a Global Molecular Network. Thirty-five total genes or group of genes are present in each network. This network characterized genes related to cellular movement, hematological system development and function and immune cell trafficking. It includes 15 genes and 4 groups from our dataset. (PDF 332 kb)

Additional file 10: Table E7. Association of the dataset #2 genes with Global Molecular Networks created by IPA using dataset 1 (Figs. E1 and E2, and not shown). (PDF 86 kb)

## Abbreviations

ECM: Extracellular matrix
EDN: Eosinophil-derived neurotoxin
FBS: Fetal bovine serum
GUSB: ß-glucuronidase
HA-IgG: Heat aggregated IgG
HLF: Primary human lung fibroblasts
IgG: Immunoglobin-G
IL-1: Interleukin-1
IL3IgG: IL-3 primed for 20 h and seeded on HA-IgG for 6 h
IPA: Ingenuity pathway analysis
rhIL3IgG: Recombinant human IL-3 plus soluble heat aggregated IgG
RNA-seq: RNA sequencing
TGF-ß: Transforming growth factor-β
