# Supplementary material for: RNA-sequencing analysis of lung primary fibroblast response to eosinophil-degranulation products predicts downstream effects on inflammation, tissue remodeling and lipid metabolism
Source: Respir Res. 2017 Nov 10;18:188. doi: 10.1186/s12931-017-0669-8 (PMC5681771; doi:10.1186/s12931-017-0669-8)
Supplement: Supplementary file 1 — Primer sequences used for real-time PCR. (DOCX 15 kb) [file 12931_2017_669_MOESM1_ESM.docx]

**Table E1:** Primer sequences used for real-time PCR

| **GENE** | **Forward primer** | **Reverse primer** |
| --- | --- | --- |
| CXCL1 | Taqman primers # Hs00236937_m1 (Life Technologies) | |
| CXCL8 | Taqman primers # Hs99999034_m1 (Life Technologies) | |
| ICAM1 | GGAGGTCACCCGCAAGGT | TGGCTTCGTCAGAATCACGTT |
| IL6 | TGCAGATGAGTACAAAAGTCCTGA | GTGGTTATTGCATCTAGATTCTTTGC |
