# Supplementary figures and images for: RNA-sequencing analysis of lung primary fibroblast response to eosinophil-degranulation products predicts downstream effects on inflammation, tissue remodeling and lipid metabolism
Source: Respir Res. 2017 Nov 10;18:188. doi: 10.1186/s12931-017-0669-8 (PMC5681771; doi:10.1186/s12931-017-0669-8)

**Figure E1**

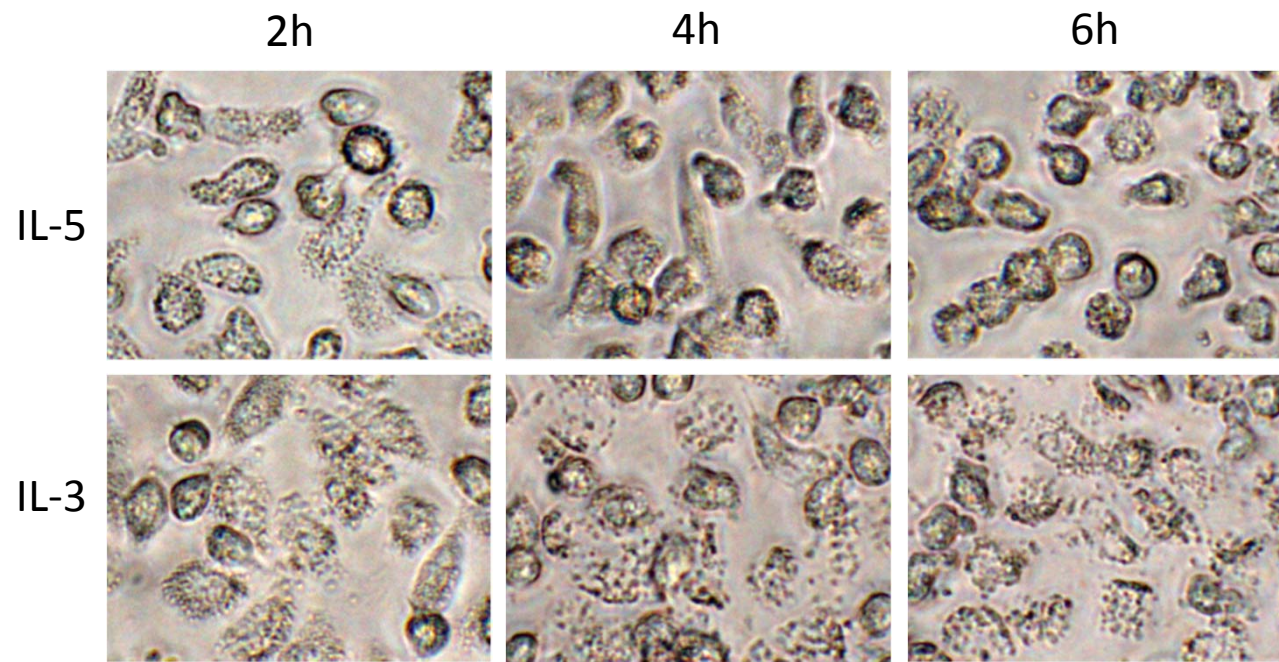

Supplement: Supplementary file 2 — IL-3-pre-activated eosinophils strongly adhere to coated HA-IgG and ultimately release free granule proteins. Human blood eosinophils were activated with IL-3 or IL-5 for 20 h and were then added on heat-aggregated (HA) human serum IgG. Photomicroscopy of IL-3 and IL-5-pre-activated eosinophils on HA-IgG was performed at 2 h, 4 h and at 6 h, using a digital camera from Olympus. (PDF 223 kb) [file 12931_2017_669_MOESM2_ESM.pdf]

# Network 1: Inflammatory Response, Development Disorder, Neurological Disease (Score 23)

Figure E2

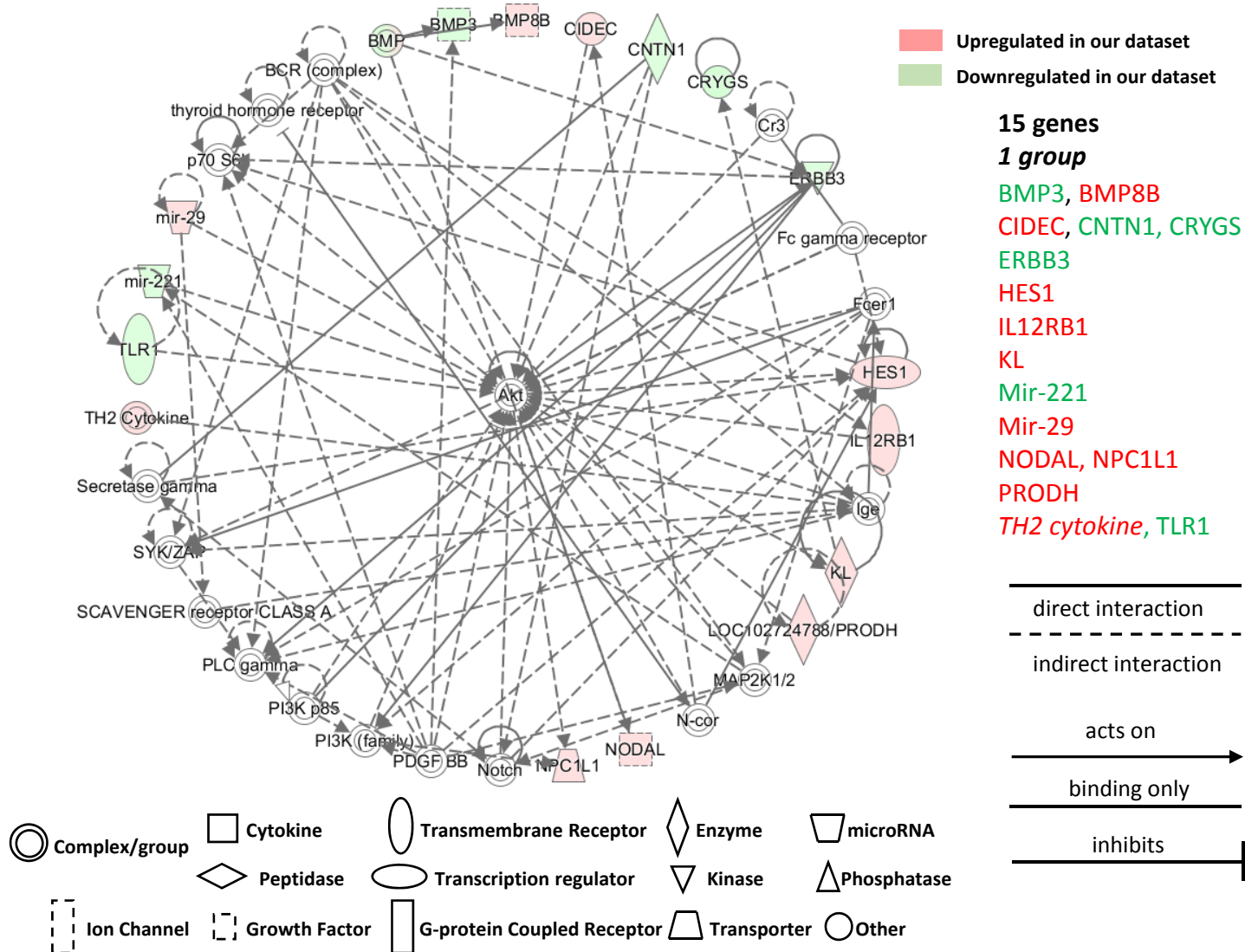

Supplement: Supplementary file 8 — Global Molecular Network #1 generated by IPA downstream analysis. Interactions between the genes of our dataset #1 (300 genes) in relation with other neighboring genes present in a Global Molecular Network. Thirty-five total genes or group of genes are present in each network. This network characterized genes related to the inflammatory response and development disorder. It includes 15 genes and 1 group from our dataset. (PDF 321 kb) [file 12931_2017_669_MOESM8_ESM.pdf]
