# Supplementary material for: RNA-sequencing analysis of lung primary fibroblast response to eosinophil-degranulation products predicts downstream effects on inflammation, tissue remodeling and lipid metabolism
Source: Respir Res. 2017 Nov 10;18:188. doi: 10.1186/s12931-017-0669-8 (PMC5681771; doi:10.1186/s12931-017-0669-8)
Supplement: Supplementary file 3 — Genes (169) up regulated by >1.5 fold in both fibroblasts lines (L20 and L21) cultured with IL3IgG eosinophil conditioned media (average of 2 eosinophil donors), compared to HLF cultured in medium only, and cultured with rhIL-3 plus HA-IgG. (PDF 88 kb) [file 12931_2017_669_MOESM3_ESM.pdf]

Table E2. Genes (169) up regulated by >1.5 fold in both fibroblasts lines (L20 and L21) cultured with IL3IgG eosinophil conditioned media (average of 2 eosinophil donors), compared to HLF cultured in medium only, and cultured with rhIL-3 plus HA-IgG.

Gene	Fold change	Gene	Fold change	Gene	Fold change	Gene	Fold change
ADAM20	7.4	ELAVL2	12.6	KYNU	3.9	RNA5S12	46.0
ADH1C	11.0	ENPEP	11.0	LGALS9B	16.7	RNA5S8	37.9
ALDH8A1	11.3	FAM124B	13.0	LHX4	2.7	RNF219-AS1	61.8
ALG1L2	2.6	FAM19A2	1.7	LYSMD2	2.0	RNU4ATAC	31.4
APOA1	12.6	FAM223B	13.4	MGAT4A	11.1	RTCA-AS1	1.9
APOL3	2.3	FAM43A	3.0	MIR1204	1.7	RTN1	9.2
ARHGAP19-SLIT1	2.1	FAM71E1	329.3	MIR29C	76.3	RTP4	3.0
ARHGEF16	9.3	FAR2P2	121.6	MIR3153	149.3	S100A14	821.9
ATP8B4	3.5	FGF10	16.7	MIR3605	6499.1	SACS-AS1	10.7
BEX2	1.7	FLJ22447	13.7	MIR3651	8808.2	SCARNA2	17.5
BMP8B	12.9	FLJ38576	1.7	MIR3661	76.3	SECTM1	2.5
BST2	15.4	FLRT1	127.9	MIR4485	7474.0	SHROOM2	1.7
C10orf82	13.3	FOXD2	100.5	MIR4649	1.8	SLC16A6	1.6
C1orf204	1.9	FOXD3	11.0	MIR6858	76.3	SLC18A2	7.6
C1QL1	15.0	FRAT1	14.4	MIR6873	6645.2	SLC1A3	175.7
C1R	2.1	GACAT2	258.4	MIR762	76.3	SLC22A14	59.9
C2orf82	19.7	GAL3ST2	10.4	MYO1H	6.4	SLC26A4-AS1	5.7
C3	59.9	GNG3	33.5	NATD1	2832.6	SLC51A	4.2
C6orf165	3.0	GPR17	11.0	NBPF4	13.3	SNORD12	37.7
C8A	13.7	GREM2	1.7	NFKBIA	1.8	SNORD38A	2105.4
CALML6	17.8	GSDMC	9.6	NFKBIZ	2.6	SNORD3B-1	1.9
CCDC102B	2.2	HAGLR	5.7	NKPD1	10.5	SNORD6	42.5
CCL2	3.7	HES1	14.4	NODAL	11.1	SORCS2	2.1
CCL4L2	278.4	HIST1H2BI	392.3	NOXRED1	13.4	SOX7	128.1
CD177	2.4	HIST2H2BE	2.0	NPC1L1	44.2	SPARCL1	14.2
CFAP46	3.2	HIST2H3D	17.7	NPTX1	2.6	SPATA6L	1.7
CH25H	4.2	IBA57-AS1	18.4	NUP210	1.7	SQSTM1	1.7
CHADL	187.2	ICAM1	3.6	OVCH1-AS1	10.1	SYNDIG1	19.2
CIDEC	328.8	IGSF6	21.9	PACERR	12.7	TAT-AS1	13.8
COL4A2-AS1	15.4	IL12RB1	11.3	PALD1	8.3	TBC1D3H	1.8
COL4A3	5.4	IL32	3.4	PAPPA-AS1	2.0	TM4SF19-AS1	1.6
CPT1B	8.7	IL6	2.3	PARD6B	10.7	TM4SF1-AS1	1.6
CXCL1	1.8	INCA1	1.8	PEG3-AS1	2.1	TMEM110-MUSTN1	10.7
CXCL8	2114.0	JAK3	151.1	PF4V1	20.2	TTC6	45.5
CYP1A1	1.7	KANK3	10.2	PLXDC1	15.2	TUBA8	1.7
CYP1B1	839.5	KCNIP2-AS1	1.9	PP14571	11.3	UCN2	1.7
DCHS2	58.7	KCNS2	9.5	PRODH	206.1	USP12-AS2	12.8
DLEU1-AS1	18.7	KHDC1L	18.2	PTPRN2	15.4	USP17L1	10.5
DLX1	15.7	KIAA2012	6.0	PYHIN1	2.7	VDR	2.2
DRICH1	2.1	KL	8.4	RAB41	384.0	VSIG10L	2.3
EFCAB8	10.0	KRT36	5.0	RAB9B	17.0	ZC3H12A	2.0
EHF	11.9	KRTAP1-3	18.6	RELB	2.0	ZMYND15	7.6
						ZNF559-ZNF177	36.6
