# Supplementary material for: RNA-sequencing analysis of lung primary fibroblast response to eosinophil-degranulation products predicts downstream effects on inflammation, tissue remodeling and lipid metabolism
Source: Respir Res. 2017 Nov 10;18:188. doi: 10.1186/s12931-017-0669-8 (PMC5681771; doi:10.1186/s12931-017-0669-8)
Supplement: Supplementary file 4 — Genes (131) down regulated by >1.5 fold in both fibroblasts lines (L20 and L21) cultured with IL3IgG eosinophil conditioned media (average of 2 eosinophil donors), compared to HLF cultured in medium only, and cultured with rhIL-3 plus HA-IgG. (PDF 88 kb) [file 12931_2017_669_MOESM4_ESM.pdf]

Table E3. Genes (131) down regulated by >1.5 fold in both fibroblasts lines (L20 and L21) cultured with IL3IgG eosinophil conditioned media (average of 2 eosinophil donors), compared to HLF cultured in medium only, and cultured with rhIL-3 plus HA-IgG.

Gene	Fold change	Gene	Fold change	Gene	Fold change	Gene	Fold change
ABCA13	-10.7	ERBB3	-19.6	MYOM1	-21.6	RRS1-AS1	-5.2
ADM5	-27.6	EXTL1	-1.6	N4BP2L2-IT2	-14.9	RUNDC3A-AS1	-1.9
AK8	-17.0	FKBP6	-410.9	NDUFV2-AS1	-33.6	SERTAD4-AS1	-21.0
ANKRD13B	-1.6	FLJ12825	-2.5	NLRC4	-11.0	SLC22A1	-272.6
ANKRD24	-3.5	FOXQ1	-8.5	NNAT	-627.6	SMIM5	-47.5
ARHGAP19	-99.1	GRHL1	-2.1	NOS1AP	-9.9	SNORA25	-925.1
ARHGAP31-AS1	-8.4	HCG17	-44.3	NSG1	-45.9	SNORA48	-21.1
ARL17A	-3.0	HIST1H2AK	-315.4	NT5C1B	-2.0	SNORD26	-1652.9
ARR3	-7.2	HIST1H2BL	-15.2	NTN3	-8.3	SNORD79	-99.0
ASB5	-5.2	HIST1H3G	-48.3	ODF3L1	-145.9	SNORD96A	-5165.4
ASMTL-AS1	-2.2	HIST1H4K	-1.9	PAK3	-17.4	SPATA1	-10.4
BMP3	-23.6	HLA-DPB1	-8.7	PILRA	-18.8	SPATA9	-8.0
BTBD18	-58.0	IMPG2	-5.0	PLCH2	-6.1	SPDYE11	-1.6
C12orf56	-1.6	JAKMIP2-AS1	-15.8	PLSCR2	-47.8	SPON1	-2.1
C16orf86	-1.7	KCNU1	-3.5	POC1B-GALNT4	-63.0	SPRY3	-5.8
C17orf104	-1.8	KIAA1731NL	-16.3	PPAPDC3	-1.6	SSBP3-AS1	-47.3
C1orf101	-6.2	KRT33A	-540.0	PPIAL4B	-1.8	TAGLN3	-1.8
C1orf132	-4.7	KRT33B	-2.5	PPIAL4F	-2.2	TAS1R3	-48.5
C5orf30	-1.7	KRTAP1-1	-2.0	PPL	-5.5	TAS2R31	-133.3
CA14	-252.4	KRTAP2-1	-320.3	PPP1R32	-1.7	TBC1D3K	-7.3
CCDC169	-2.2	KY	-7.8	PPP1R3G	-9.0	TCEAL5	-8.0
CHDH	-213.3	LGALSL	-5.4	PRKACG	-15.8	TLR1	-4.6
CNTN1	-3.4	LILRB3	-15.8	PRR7	-1.7	TMC3	-4.1
CRYGS	-923.4	LRGUK	-6.5	PRRG4	-3.8	TNFRSF25	-71.8
CXCR6	-6.5	LRRN4	-7.3	PTPRR	-1.6	TOLLIP-AS1	-1.8
DCC	-1.8	M1AP	-88.0	RAET1E	-1.8	TPPP3	-2.5
DDN	-22.0	MANEA-AS1	-304.8	RASSF1-AS1	-61.0	TRPC5	-212.1
DNAH10	-2.4	MAST1	-4.8	RIBC1	-6.6	WBSCR27	-1.5
DRD1	-40.1	MBP	-1.8	RNF180	-1.9	WWTR1-AS1	-1.8
EGR3	-10.3	MEX3B	-1.9	RNU6-36P	-167.3	ZASP	-4.6
EHHADH-AS1	-1.9	MIR221	-106.7	RNU6-42P	-28.7	ZNF540	-33.4
ELFN2	-4.2	MIR7110	-11269.9	RNU6-7	-115.9	ZNF575	-2.5
EPHX4	-227.7	MYO5C	-2.8	RORB	-1.8		
