# Supplementary material for: RNA-sequencing analysis of lung primary fibroblast response to eosinophil-degranulation products predicts downstream effects on inflammation, tissue remodeling and lipid metabolism
Source: Respir Res. 2017 Nov 10;18:188. doi: 10.1186/s12931-017-0669-8 (PMC5681771; doi:10.1186/s12931-017-0669-8)
Supplement: Supplementary file 5 — Upstream Regulators of the genes listed in our dataset (target genes, Additional file 3: Table E2 and Additional file 4: Table E3) as predicted by IPA analysis. (PDF 141 kb) [file 12931_2017_669_MOESM5_ESM.pdf]

Table E4. Upstream Regulators of the genes listed in our dataset (target genes, Table E2 and E3) as predicted by IPA analysis.

Upstream Regulator	*P* value of overlap	Activation z-score	Target molecules in dataset	
			**Genes upregulated in dataset**	**Genes downregulated**
**Cytokine**	Target molecules in dataset (≥ 10 genes)			
**IFNG**	1.10 10^{-3}	2.803	BST2, C1R, C3, CCL2, CH25H, CXCL1, CXCL8, EHF, ELAVL2, ICAM1, IL12RB1, IL32, IL6, JAK3, KYNU, LGALS9B, NFKBIA, NFKBIZ, RTP4, SLC1A3, SQSTM1, VDR (22)	EGR3, FKBP6, TLR1
**IL1A**	7.79 10^{-5}	2.567	CCL2, CXCL1, CXCL8, CYP1A1, ICAM1, IL32, IL6, NFKBIA, NFKBIZ, ZC3H12A (10)	
**IL4**	5.55 10^{-2}	2.275	C3, CCL2, CXCL1, CXCL8, ICAM1, IL12RB1, IL6, JAK3, NFKBIA, SQSTM1, USP17L1, VDR (12)	LGALSL
**CSF2**	6.77 10^{-4}	1.999	C3, CCL2, CXCL1, CXCL8, ICAM1, IL32, IL6, NFKBIA, RELB, SQSTM1, VDR (11)	EGR3, TLR1
**Signaling**	Target molecules in dataset (Activation z-score > 2.220)			
**TICAM1**	*1.08 10^{-5}*	*2.417*	CCL2, CH25H, CXCL8, ICAM1, IL6, NFKBIA, NFKBIZ, RELB	PILRA
**TRADD**	*3.08 10^{-7}*	*2.414*	CCL2, CXCL1, CXCL8, ICAM1, IL6, NFKBIA	
**ERK1/2**	*6.67 10^{-2}*	*2.384*	C3, CCL2, CXCL1, CXCL8, ICAM1, IL6	
**IKBKB**	*1.22 10^{-5}*	*2.230*	C3, CCL2, CH25H, CXCL1, CXCL8, CYP1B1, FGF10, ICAM1, IL6, RELB, LGALS9B, NFKBIA (12)	MBP
**Kinase Inhibitors**	Target molecules in dataset (activation z-score < -2.300)			
**SB203580**	*2.77 10^{-4}*	*-3.176*	BST2, C3, CCL2, CXCL1, CXCL8, CYP1A1, CYP1B1, EHF, ICAM1, IL6, SECTM1, VDR	
**PD98059**	*2.29 10^{-2}*	*-2.774*	APOA1, C3, CCL2, CXCL1, CXCL8, CYP1A1, CYP1B1, ICAM1, IL6, VDR	EGR3
**PS-1145**	*2.39 10^{-8}*	*-2.418*	CCL2, CXCL1, CXCL8, ICAM1, IL6, NFKBIA	
**LY294002**	*4.78 10^{-3}*	*-2.412*	ADH1C, CCL2, CXCL1, CXCL8, HES1, ICAM1, IL32, IL6, NFKBIA, NPTX1	EGR3, ERBB3
**Transcription Factor**	Target molecules in dataset (Activation z-score > 2.200)			
**CEBPA**	*6.15 10^{-2}*	*2.400*	ADH1C, C3, CXCL8, ICAM1, IL6, SECTM1, VDR	PPL
**EGR1**	*3.12 10^{-2}*	*2.213*	APOA1, CCL2, CXCL8, ENPEP, ICAM1	
**Others**				
**Lipopolysaccharide (chemical drug)**	*4.89 10^{-3}*	*3.591*	APOA1, C3, CCL2, CH25H, CPT1B, CXCL1, CXCL8, CYP1A1, EHF, HES1, ICAM1, IL32, IL6, IL12RB1, IGSF1, JAK3, KYNU, NFKBIA, NFKBIZ, NPTX1, RELB, SECTM1, VDR,	DDN, EGR3, SLC22A1, TLR1
**Poly rI:rC-RNA (Biologic drug)**	*7.03 10^{-6}*	*3.322*	BST2, C3, CCL2, CXCL1, CXCL8, DLX1, EHF, ELAVL2, ICAM1, IL32, IL6, LGALS9B, RELB, KYNU, NFKBIA	TLR1
**APP (other)**	*1.35 10^{-2}*	*3.199*	C1R, C3, CCL2, CH25H, CXCL1, CXCL8, HES1, ICAM1, IL6, KL, KYNU, NPTX1, RTP4, TUBA8	MBP, PAK3,
**Tretinoin (endogenous mammalian)**	*2.68 10^{-1}*	*2.603*	C3, CCL2, CCL4L1, CPT1B, CXCL8, CYP1A1, FLRT1, FOXD3, HES	CCDC169
**F2 (peptidase/thrombine)**	*1.15 10^{-3}*	*2.498*	CCL2, CXCL1, CXCL8, ICAM1, IL32, IL6, NFKBIA, RELB	TAGLN3
