# Supplementary material for: RNA-sequencing analysis of lung primary fibroblast response to eosinophil-degranulation products predicts downstream effects on inflammation, tissue remodeling and lipid metabolism
Source: Respir Res. 2017 Nov 10;18:188. doi: 10.1186/s12931-017-0669-8 (PMC5681771; doi:10.1186/s12931-017-0669-8)
Supplement: Supplementary file 6 — Genes downregulated by IL3IgG- versus IL5IgG-activated eosinophil conditioned medium and control media (medium only and rhIL3IgG) in fibroblasts. (PDF 94 kb) [file 12931_2017_669_MOESM6_ESM.pdf]

Table E6. Genes downregulated by IL3IgG- versus IL5IgG-activated eosinophil conditioned medium and control media (medium only and rhIL3IgG) in fibroblasts.

Gene	Protein	Brief description	Regulators-Networks (IPA analyses)
**ABCA13**	ATP-binding cassette, sub-family A (ABC1), member 13	Transmembrane movement	Network 2: cellular movement (Figure E2)
**BMP3 (secreted)**	bone morphogenetic protein 3	Bind TGF-ß receptors- Suppresses TGF-ß receptor availability	Network 1: development disorder (Figure E1)
**BTBD18**	BTB (POZ) domain containing 18	Protein ubiquitination	
**CA14**	carbonic anhydrase XIV	Reversible hydration of CO2	
**CAPSL**	calcyphosine-like	Calcium ion binding	
**CCDC169**	coiled-coil domain containing 169		
**CHDH**	choline dehydrogenase	Amine and ployamine biosynthesis	
**DCC**	DCC netrin 1 receptor	Axon guidance- FAK pathway- Induces apoptosis- Tumor suppressor	
**DDN**	dendrin	Promote apoptosis and polymerase II activity	
**DRD1**	dopamine receptor D1	Activates cAMP/AMPK pathways- Neuronal growth	
**ERBB3 (secreted)**	v-erb-b2 avian erythroblastic leukemia viral oncogene homolog 3	Inhibits or activates neuregulin signal	Target molecule (Table 2); Regulated by OSM, Akt (Table 3); Networks: immune response (inhibitor); Network 1: development disorder, neurological disease
**GPR3**	G protein-coupled receptor 3	Activates cAMP- APP generation	
**INAFM2**	InaF-motif containing 2		
**KRT33A**	keratin 33A, type I	Hair and nail formation	
**LGALSL**	lectin, galactoside-binding-like		
**MBP**	myelin basic protein	Signaling in T-cells and neuronal cells	Target molecules (Table 2); Regulated by JUN, IKBKB, APP (Tables 3 and E3)
**MGAT5B**	mannosyl (alpha-1,6-)-glycoprotein beta-1,6-N-acetyl-glucosaminyltransferase, isozyme B	Neurite outgrowth by integrin interaction with ECM proteins	
**NLRC4**	NLR family, CARD domain containing 4	Part of the inflammasome	
**NNAT**	neuronatin	Nervous system development- Regulatory subunit of ion channels	
**NSG1**	neuron specific gene family member 1	Modulates amyloidogenic processing	
**ODF3L1**	outer dense fiber of sperm tails 3-like 1		
**PDE6G**	phosphodiesterase 6G, cGMP-specific, rod, gamma	Regulates c-Src protein kinase and GRK2	
**PLCH2**	phospholipase C, eta 2	Formation of IP3, DAG, and the neuronal network	
**PLSCR2**	phospholipid scramblase 2	Role in coagulation and apoptosis	
**PPIAL4F**	peptidylprolyl isomerase A (cyclophilin A)-like 4F		
**PPL**	periplakin	Part of Akt1-mediated signaling	Regulated by CEBPA (Table E3)
**RAET1E**	retinoic acid early transcript 1E	Ligand for NKG2D- Role in the immune response	
**RIBC1**	RIB43A domain with coiled-coils 1		
**SLC22A1**	solute carrier family 22 (organic cation transporter)	Inhibited by PKA, activated by calmodulin complex	Networks: lipid metabolism
**SPATA1**	spermatogenesis associated 1		
**SPATA9**	spermatogenesis associated 9		
**SPON1 (secreted)**	spondin 1, extracellular matrix protein	Cell adhesion- Major factor for vascular smooth muscle cell growth	Network 2: cellular movement
**SPRY3**	sprouty homolog 3 (Drosophila)	Antagonist of fibroblast growth factor pathways- Negatively modulate respiratory organogenesis	
**TAS2R31**	taste receptor, type 2, member 31		
**TLR1**	toll-like receptor 1	Pathogen recognition and activation of innate immunity- Acts via MYD88	Networks: immune response. Network 1: inflammatory response
**TMC3**	transmembrane channel-like 3		
**ZNF575**	zinc finger protein 575		
