# Supplementary material for: RNA-sequencing analysis of lung primary fibroblast response to eosinophil-degranulation products predicts downstream effects on inflammation, tissue remodeling and lipid metabolism
Source: Respir Res. 2017 Nov 10;18:188. doi: 10.1186/s12931-017-0669-8 (PMC5681771; doi:10.1186/s12931-017-0669-8)
Supplement: Supplementary file 7 — HLF (L20 and L21) expression levels for the receptors of the upstream regulators identified by IPA (Tables 2 and 3, and Additional file 4: Table E3), as determined by our RNA-seq analysis. (PDF 92 kb) [file 12931_2017_669_MOESM7_ESM.pdf]

Table E5. HLF (L20 and L21) expression levels for the receptors of the upstream regulators identified by IPA (Tables 2 and 3, and Table E3), as determined by our RNA-seq analysis.

Gene	Level of expression in HLF (RNA-seq/ log2 total RPKM)			
	L20		L21	
	Medium	IL3IgG	Medium	IL3IgG
**Receptors for the upstream regulators**				
**TNFRSF1A (TNF-α receptor)**	**6.46**	**6.7**	**6.81**	**6.9**
**IL1R1 (IL1 receptor)**	**3.82**	**3.9**	**4.73**	**4.6**
**IL1RAP (IL1 receptor)**	**2.47**	**3.1**	**1.53**	**1.9**
**OSMR (OSM type 2 receptor)**	**5.28**	**5.5**	**5.51**	**5.9**
**LIFR (OSMR type 1 receptor)**	**3.48**	**3.0**	**3.46**	**3.0**
**IL6ST (OSM, IL27 and IL6 commun-chain receptor)**	**5.93**	**5.9**	**6.51**	**6.3**
**IL6R (IL6 receptor)**	**1.55**	**1.6**	**2.84**	**3.1**
**IFNGR1 (IFNG receptor)**	**4.76**	**4.7**	**4.24**	**4.6**
**IFNGR2 (IFNG receptor)**	**5.30**	**5.4**	**5.58**	**5.8**
**IL4R (IL4 and IL13 commun receptor)**	**4.36**	**4.5**	**4.42**	**4.9**
IL2RG (IL4, IL2, IL7 and IL21 γ-chain receptor)	-10.00	-10.0	-8.00	-8.0
**IL13RA1 (IL13 and IL4 commun receptor)**	**5.53**	**5.5**	**6.11**	**5.8**
CSF2RA (GM-CSF receptor)	-10.00	-10.0	-8.00	-8.0
CSF2RB (GM-CSF, IL-5, IL-3 ß-chain receptor)	-10.00	-10.0	-8.00	-8.0
**IL17RA (IL17A and IL17F receptor)**	**3.13**	**3.2**	**3.69**	**3.7**
**IL17RC (IL17A and IL17F receptor)**	**3.67**	**3.5**	**3.30**	**3.5**
**IL27RA (IL27 receptor)**	**-1.54**	**-1.4**	**0.10**	**-0.2**
IL18R1 (IL18 receptor)	-10.00	-10.0	-8.00	-2.8
IL18RAP (IL18 receptor)	-10.00	-10.0	-8.00	-8.0
**IFNAR1 (IFNA receptor)**	**4.48**	**4.2**	**4.53**	**4.9**
**IFNAR2 (IFNA receptor)**	**2.83**	**2.9**	**3.31**	**3.4**
**TNFRSF12A (FN14)**	**7.35**	**7.2**	**6.91**	**7.7**
**C5AR1 (C5)**	**-1.84**	**-1.7**	**-0.63**	**-1.6**
IL32 receptor (unknown)				
FGA, FGB, FGG (F2/thrombin receptor)	-10.00	-10.0	-8.00	-8.0
**F2R/PAR1 (F2/thrombin receptor)**	**7.97**	**7.8**	**7.31**	**7.3**
**F2RL2/PAR3 (F2/thrombin receptor)**	**4.34**	**3.8**	**3.10**	**3.5**
**F2RL1/PAR2 (F2/thrombin receptor)**	**2.49**	**2.5**	**1.31**	**1.6**
F2RL3 (F2/thrombin receptor)	-10.00	-10.0	-8.00	-8.0
**Upstream regulators expressed in HLF**				
**IL6**	**0.44**	**1.6**	**1.09**	**3.3**
**TNFSF12**	**3.05**	**3.5**	**3.87**	**4.1**
**C5**	**0.45**	**0.7**	**1.55**	**1.2**
**IL32**	**-1.32**	**0.4**	**2.11**	**4.1**
**Other upstream receptors or secreted proteins**				
**SELPLG (SELP ligand)**	**1.35**	**1.8**	**1.82**	**2.2**
**APP**	**8.91**	**9.1**	**9.01**	**8.9**
**TLR4**	**-1.35**	**-1.7**	**-4.31**	**-2.1**
**VCAN**	**5.93**	**5.8**	**6.02**	**6.1**
**EGR1**	**2.73**	**3.0**	**2.85**	**3.2**
**FN1**	**12.83**	**13.0**	**11.52**	**11.0**
**HMGB1**	**6.19**	**6.2**	**6.25**	**6.4**
**HMOX1**	**7.74**	**8.0**	**6.17**	**6.6**

Gene expression in HLF cultured for 24 h with (IL3IgG) or without (medium) IL3IgG conditioned medium from eosinophils. IL3IgG is an average of 2 eosinophil donors
