# Supplementary material for: RNA-sequencing analysis of lung primary fibroblast response to eosinophil-degranulation products predicts downstream effects on inflammation, tissue remodeling and lipid metabolism
Source: Respir Res. 2017 Nov 10;18:188. doi: 10.1186/s12931-017-0669-8 (PMC5681771; doi:10.1186/s12931-017-0669-8)
Supplement: Supplementary file 9 — Global Molecular Network #2 generated by IPA downstream analysis. Interactions between the genes of our dataset #1 (300 genes) in relation with other neighboring genes present in a Global Molecular Network. Thirty-five total genes or group of genes are present in each network. This network characterized genes related to cellular movement, hematological system development and function and immune cell trafficking. It includes 15 genes and 4 groups from our dataset. (PDF 332 kb) [file 12931_2017_669_MOESM9_ESM.pdf]

Figure E3

Network 2: Cellular Movement, Hematological System Development and Function, Immune Cell Trafficking (Score 23)

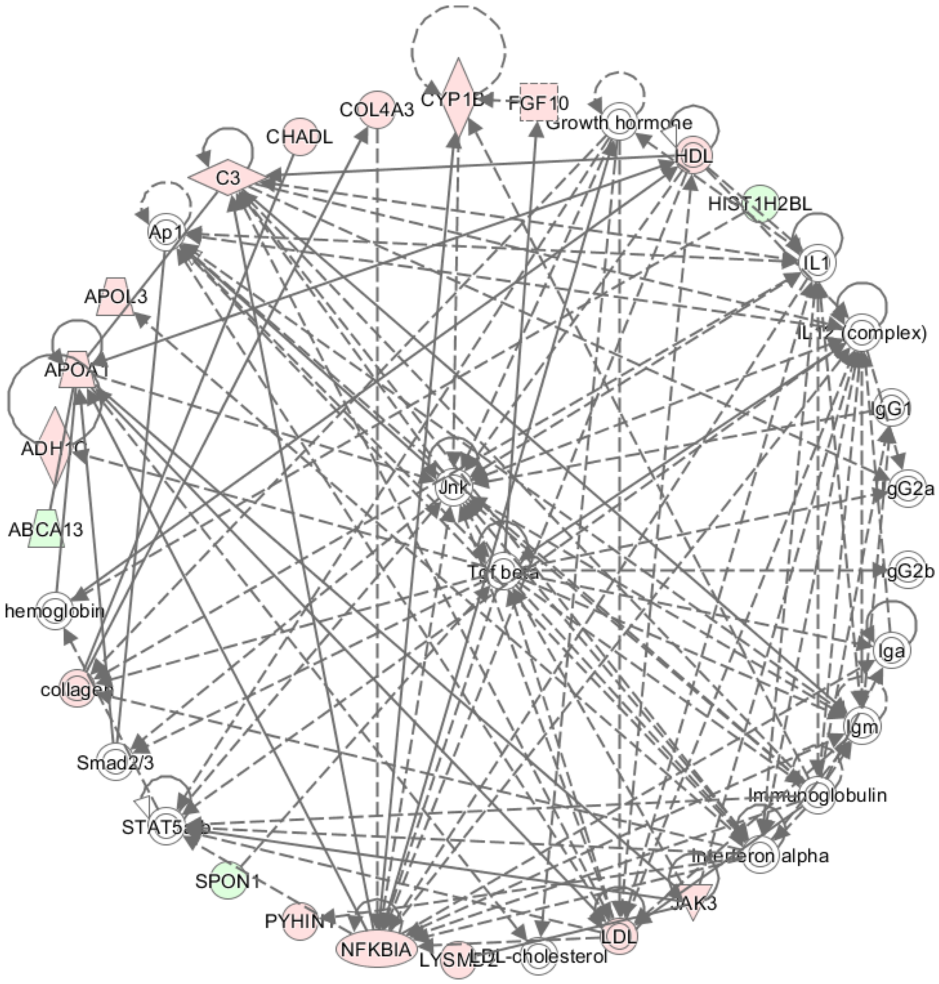

15 genes
4 groups

ABCA13, ADH1C, APOA1, APOL3
C3, CHADL, COL4A3, *collagen*, CYP1B1
FGF10
HDL, HIST1H2BL
JAK3
LDL, LYSMD2
NFKBIA
PYHIN1
SPON1

Upregulated in our dataset
Downregulated in our dataset

direct interaction
indirect interaction
acts on
binding only
inhibits

Complex/group **Cytokine** **Transmembrane Receptor** **Enzyme** **microRNA**
Peptidase **Transcription regulator** **Kinase** **Phosphatase**
Ion Channel **Growth Factor** **G-protein Coupled Receptor** **Transporter** **Other**
