# Supplementary material for: RNA-sequencing analysis of lung primary fibroblast response to eosinophil-degranulation products predicts downstream effects on inflammation, tissue remodeling and lipid metabolism
Source: Respir Res. 2017 Nov 10;18:188. doi: 10.1186/s12931-017-0669-8 (PMC5681771; doi:10.1186/s12931-017-0669-8)
Supplement: Supplementary file 10 — Association of the dataset #2 genes with Global Molecular Networks created by IPA using dataset 1 (Figs. E1 and E2, and not shown). (PDF 86 kb) [file 12931_2017_669_MOESM10_ESM.pdf]

Table E7. Association of the dataset #2 genes with Global Molecular Networks created by IPA using dataset 1 (Figures E1 and E2, and not shown)

Global Molecular Network (IPA)	Genes	
	Upregulated	Downregulated
**1. Inflammatory Response**	**BMP8B, CIDEC, PRODH**	**BMP3, ERBB3, TLR1**
**2. Cellular Movement**	**C3, CH25H, PYHIN1**	**ABCA13, SPON1**
**3. Cell Cycle**	**FAM124B, KRT36**	**CHDH, SPRY3**
**4. Skeletal and Muscular System Development and Function**	**CXCL1, CXCL8, NFKBIZ, UCN2**	**DCC**
**5. Development Disorder**	**CH25H, CPT1B, ZC3H12A**	**SLC22A1**
**6. Cell Morphology**	**CYP1A1, FOXD3**	**MBP**
